# Morphological characteristics of pollen from triploid watermelon and its fate on stigmas in a hybrid crop production system

**DOI:** 10.1038/s41598-022-06297-2

**Published:** 2022-02-25

**Authors:** Erandi C. W. Subasinghe Arachchige, Lisa J. Evans, Ulrika Samnegård, Romina Rader

**Affiliations:** 1 School of Environmental and Rural Science, University of New England, Armidale, NSW 2351 Australia; 2 Plant and Food Research Australia, c/o Queensland University of Technology, Brisbane, QLD Australia; 3 Department of Horticulture and Landscape Gardening, Faculty of Agriculture and Plantation Management, Wayamba University of Sri Lanka, Makandura, Gonawila, 60170 North Western Province Sri Lanka; 4 Department of Biology, Lund University, 223 62 Lund, Sweden

**Keywords:** Plant sciences, Ecology, Environmental sciences

## Abstract

Hybrid crop production is more reliant on pollinators compared to open-pollinated crops because they require cross-pollination between a male-fertile and a male-sterile line. Little is known about how stigma receipt of pollen from male-sterile genotypes affects reproduction in hybrids. Non-viable and non-compatible pollen cannot fertilise plant ovules, but may still interfere with pollination success. Here we used seedless watermelon (*Citrullus lanatus* (Thunb.) Matsum. & Nakai) as a model hybrid plant, to evaluate the morphology, physiology, and movement of pollen from inter-planted genotypes (diploids and triploids). We found that pollen from triploids (‘Exclamation’ and ‘Royal Armada’) and diploids (‘SP-6’, ‘Summer Flavor 800’, and ‘Tiger’) was visually distinguishable. Pollen in triploids had more deformities (42.4–46%), tetrads (43–44%), and abnormal growth of callose plugs in pollen tubes. The amount of pollen in triploids to germinate on stigmas was low (8 ± 3%), and few pollen grains produced pollen tubes (6.5 ± 2%). Still, contrary to previous reports our results suggest that some viable pollen grains are produced by triploid watermelons. However, whilst honey bees can collect and deposit pollen from triploids onto stigmas, its effect on hybrid watermelon reproduction is likely to be minimal due to its low germination rate.

## Introduction

Many economically important food and seed crops, including tomato, sweet pepper, cucurbits, carrots, and brassicas, are now grown in commercial fields as hybrids^1^. Hybrid crops are produced by crossing selected parental lines, with the objective of producing offspring that have superior genetic characteristics from both parental lines (i.e. hybrid vigour) and generally produce greater yield quantity and/or quality characteristics^2,3^. This form of production is more reliant on pollinators compared to open-pollinated crops as one of the parental lines in hybrid production is functionally male-sterile (i.e. does not produce viable and/or compatible pollen grains) and requires insects to move pollen from male-fertile lines^4^.

Understanding the characteristics of pollen and its transfer from different parental lines/ genotypes is important because several conditions can influence the quality of pollen on stigmas and thereby affect crop production^5^. At the pre-pollination stage, suboptimal cross-pollination can occur when there are inherent physiological differences among the parental lines^6^. For example, pollinator visitation rates can be low^7^ and/or pollinator movement between lines can be limited^8,9^ when the flowers produced by different hybrid lines are not equally attractive to pollinators (e.g. onion and seed potato^7,10^), or when anthesis of parental lines does not overlap^4^.

Post-pollination interactions (i.e. pollen–pistil and/or pollen–pollen interactions) are also fundamental to the success of hybrid production. Once an insect has visited a flower, an adequate number of pollen grains need to germinate on the stigmatic surface and develop pollen tubes, in order for fertilisation to take place and seeds/fruit to develop. Successful fertilisation requires stigmas to be receptive and pollen to be both viable and compatible^11,12^. The flowers of male-sterile plants can produce abnormal pollen, often with assumed low viability^13,14^. Yet for many crops, the physiology of the pollen grains produced by male-sterile flowers has not been quantified and little is known about how the transfer of this pollen onto receptive stigmas affects pollination outcomes.

Pollen that is not viable or compatible cannot fertilise plant ovules, however such pollen may negatively affect pollination success through mechanical clogging and/or inhibition of pollen tube growth of viable pollen^15,16^. In hybrid production, the availability of non-viable pollen in the production area can be similar or greater than the amount of viable pollen, and can be removed by pollinators at a comparable rate^17,18^. Hence, there is potential for high numbers of non-viable pollen to be deposited on receptive stigmas, with possible negative effects upon plant reproduction.

In this study, we used seedless watermelon as a model hybrid crop species to compare the properties of pollen from triploid (male sterile line) and diploid (male fertile line) flowers and to investigate the fate of pollen produced by triploid flowers in commercial fields.

We ask the following research questions:

(i) Are pollen grains produced by triploids morphologically different and hence distinguishable from pollen grains produced by diploid flowers and how do both genotypes vary among cultivars?
(ii) Do differences in pollen-pistil interactions (i.e. triploid x diploid vs triploid x triploid) impact the germination of the pollen to the stigma?
(iii) What proportion of the different pollen grains from diploid and triploid lines are transferred by foraging honey bees?
(iv) What is the outcome of pollination success (measured as pollen tube growth) as a result of the genotype of the donor and recipient crosses?

## Results

### Pollen morphology

**Figure 1:**
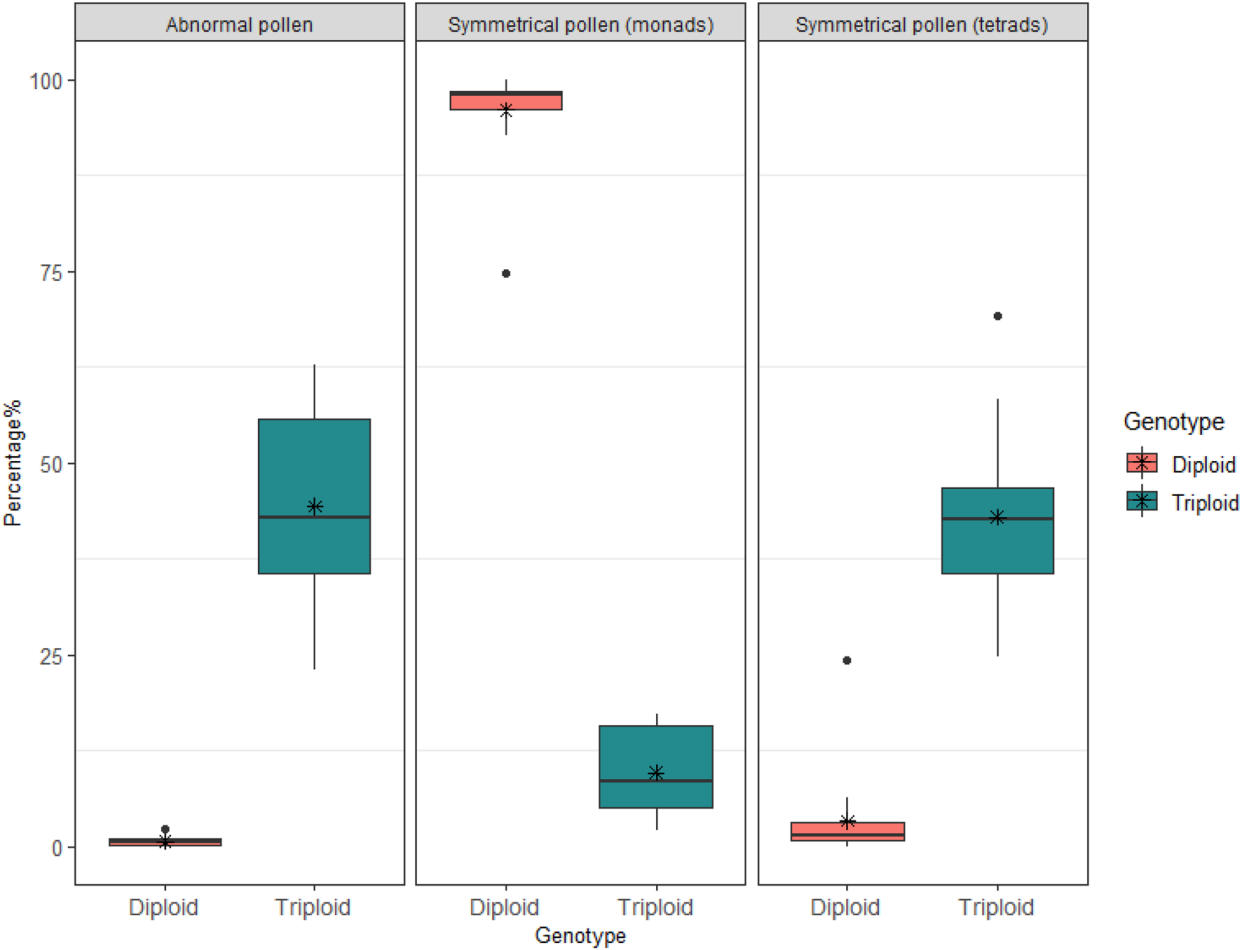
Comparison of pollen grain characteristics of triploid and diploid watermelon genotypes, including the percentage (per flower) of abnormal pollen, and symmetrical pollen as tetrads and monads. Each box indicates quartiles with median and points outside of the box are outliers. Mean of percentage in each genotype is marked as an asterisk (*)**.**

**Figure 2:**
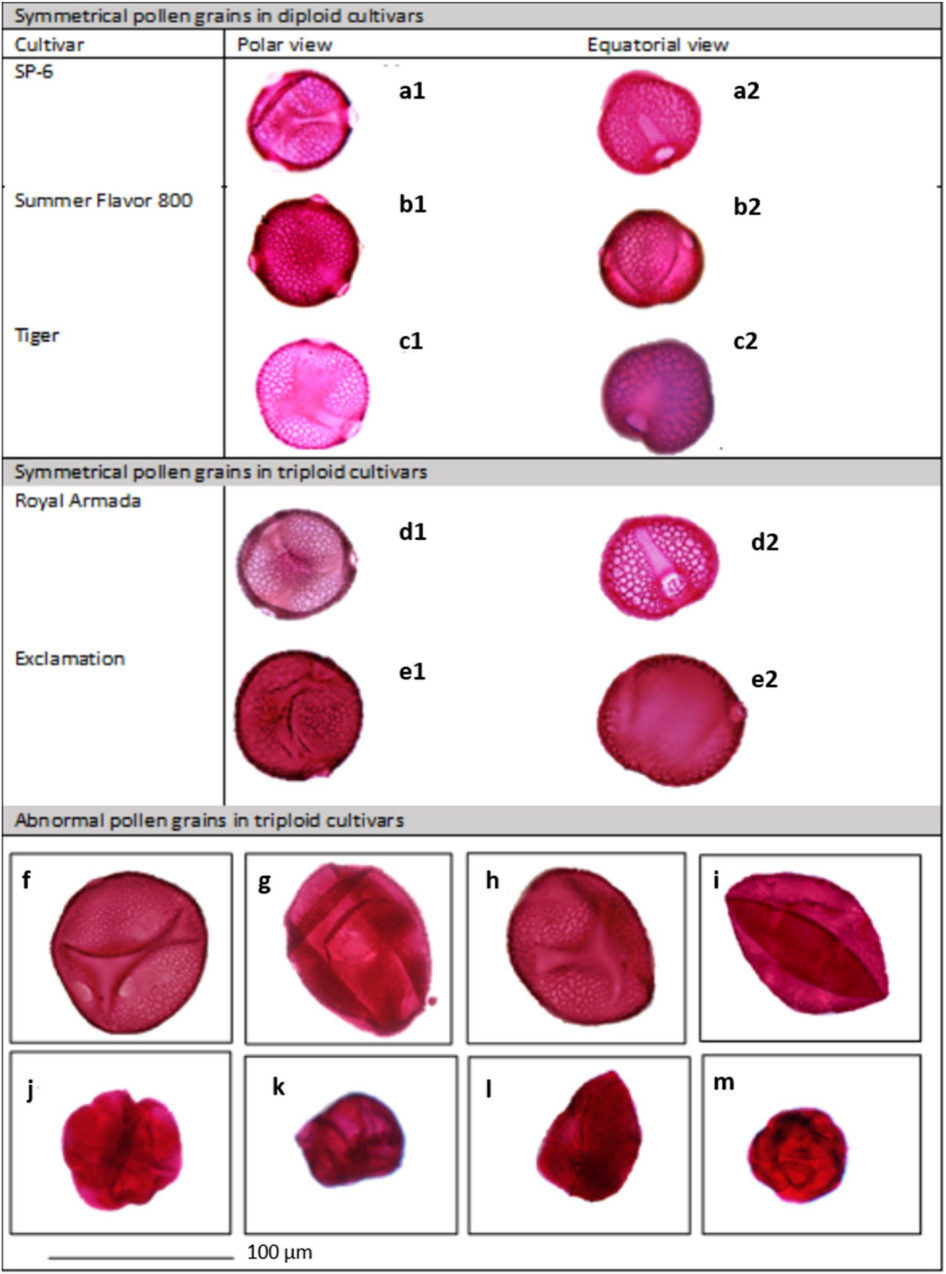
Polar view and equatorial view of symmetrical pollen grains in diploid and triploid watermelon cultivars: (**a1**,**a2**) SP-6, **(b1,b2)** ‘Summer Flavor 800’, **(c1,c2)** ‘Tiger’, **(d1,d2)** ‘Royal Armada’ and **(e1,e2)** ‘Exclamation’ and different type of abnormal pollen grains found in triploid cultivars **(f–m)**; large size irregular structures **(f–i)** and irregular shape solid structures **(j–m)**.

Pollen grains from triploid cultivars were clearly distinguishable from those of diploid cultivars (Fig. 1). We identified four main differences in morphology including: number of symmetrical monads, number of tetrads, abnormalities, and pollen size. The pollen grains from both genotypes had a characteristic tricolporate (three pori and three colpi) shape with a circular outline in both polar and equator views (Fig. 2 and Supplementary Fig. S1). However, the percentage (per flower) of symmetrically shaped monads was variable between genotypes (Fig. 1).

All diploid cultivars had a higher percentage of symmetrical pollen grains (percentage of pollen grains ± SE per flower: 96 ± 1.4%) compared to the triploid cultivars of ‘Exclamation’ (11 ± 2.5%) and ‘Royal Armada’ (8.2 ± 1.6%) (Table 1). Pollen from triploid cultivars were also more likely to be in a tetrad (Supplementary Fig. S2) or abnormally shaped (Fig. 2). Tetrads comprised 43 ± 3.25% of pollen from triploid cultivars, while the pollen of diploid cultivars contained very few tetrads (1.8 ± 0.7–6.2 ± 3.4%). Abnormalities occurred 44.4 ± 4.2% of pollen from triploid cultivars compared to < 1% of pollen in diploid cultivars (Table 1). These pollen abnormalities included large irregular-shaped pollen grains (> 100 μm) (Fig. 2f–i), pollen grains with less developed exine characters, smooth surfaces, or irregular-shaped solid structures (Fig. 2j–m, Supplementary Fig. S3).

**Table 1:** Pollen grain characteristics of five watermelon cultivars as the percentage of abnormal pollen, symmetrical pollen as tetrads and monads per flower (percentage ± SE).

Genotype	Cultivar	% Symmetrical pollen (monads)	% Symmetrical pollen (tetrads)	% Abnormal pollen
Diploid	SP-6	93 ± 3.4	6.2 ± 3.4	0.86 ± 0.13
	Summer Flavor 800	98 ± 0.9	1.8 ± 0.7	0.56 ± 0.33
	Tiger	97 ± 0.7	2 ± 0.8	0.4 ± 0.1
Triploid	Royal Armada	8.2 ± 1.6	44 ± 5.2	42.4 ± 6
	Exclamation	11 ± 2.5	43 ± 4.3	46 ± 4

Pollen size varied among cultivars [surface area (F_4, 25_ = 32.26), perimeter (F_4, 25_ = 26.2), polar axis (P) (F_4, 25_ = 28.8), equator diameter (E) (F_4, 25_ = 34.65), P < 0.001, Supplementary Table S1 and S2] and genotypes [surface area (F_1, 28_ = 5.94), perimeter (F_1, 28_ = 6.91), E (F_1, 28_ = 5.6), *P* < 0.05, Supplementary Table S2]. ‘Exclamation’ (3n) had the largest pollen grains which were significantly larger than ‘Royal Armada’ (3n) but not ‘Tiger’ (2n) pollen. ‘Royal Armada’ (3n) and other diploid cultivars ‘Summer Flavor 800’ and SP-6 were not significantly different in pollen size (Supplementary Table S3). In addition, size of other pollen morphological traits (pori and colpi) also differed among cultivars (Supplementary Table S3) but not between genotypes (Supplementary Fig. S4) except pori width. The ratio between colpi length and P was not significantly different between genotypes or among cultivars (Supplementary Table S1, S2 and S3).

All the assessed watermelon cultivars produced medium size pollen grains (considered only symmetrical pollen grains: 64–75 μm) according to previous classification^19^. All the cultivars displayed higher E than P while P/E varied from 0.96 to 0.99 and it was not significantly different between genotypes or among cultivars. Hence, all the cultivars produced oblate-spheroidal shape pollen grains^19^.

### Pollen-pistil interaction: triploid × diploid and triploid × triploid crosses

#### Hand-pollination

**Figure 3:**
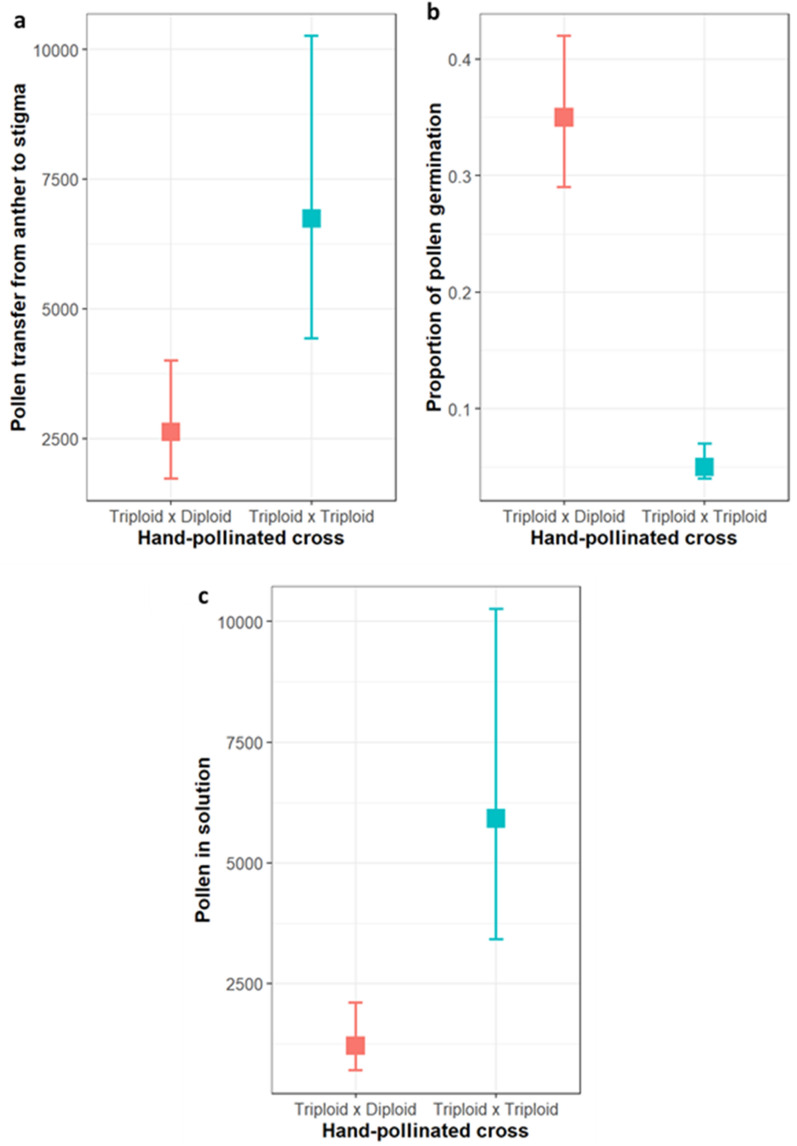
Number of pollen grains **(a)** transferred from anthers to stigma and **(b)** proportion of pollen germination on stigma **(c)** in the solution (ethanol and NaOH) in two hand-pollinated crosses; triploid × diploid cross (n = 8) and triploid × triploid cross (n = 8). Estimated means (square mark) and 95% confidential intervals are back-transformed from the logit scale.

Significantly more pollen was transferred onto triploid stigmas after being brushed with triploid anthers (mean ± SE: 7772 ± 1853 pollen grains), compared to diploid anthers (3032 ± 572 pollen grains) (model estimate = 0.94, SE = 0.01, *P* < 0.001, Fig. 3a). However, the proportion of pollen that germinated on the stigmatic surface (i.e., pollen on the stigma, excluding pollen suspended in the solutions) was significantly higher when stigmas received pollen from diploids (60 ± 8% germinated) compared to triploids (8 ± 3% germinated) (model estimate = −2.32, SE = 0.02, *P* < 0.001, Fig. 3b). Conversely, more pollen grains remained in the solutions when stigmas were pollinated with pollen in triploids (model estimate = 1.58, SE = 0.01, *P* < 0.001, Fig. 3c) and 71 ± 4.5% of these pollen grains were abnormally large or in tetrads, compared to only 19 ± 5.6% in diploid solutions.

#### Pollen deposition by honey bees

**Figure 4:**
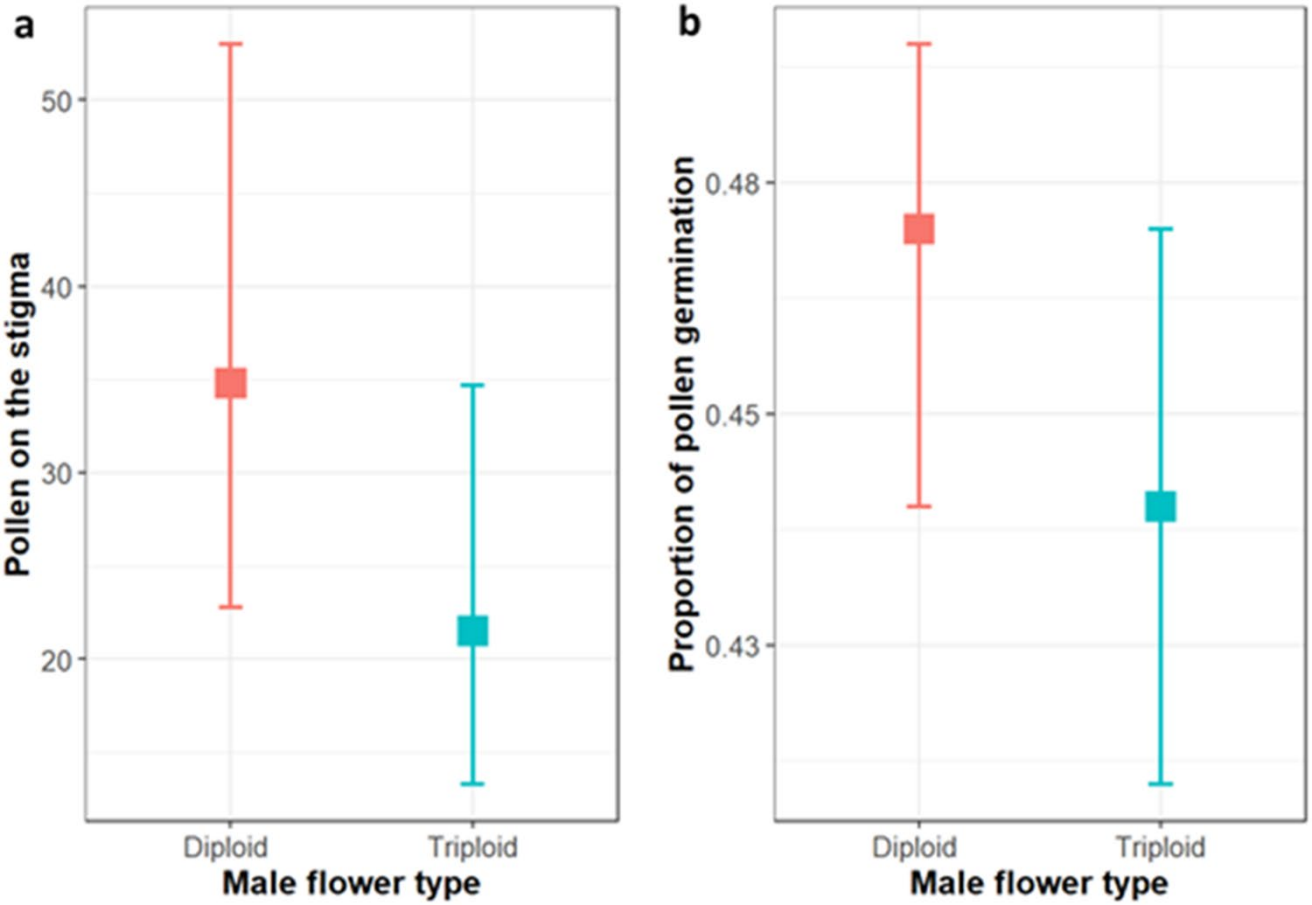
Number of pollen grains **(a)** on stigma and **(b)** proportion of pollen germination on stigma in triploid female flower followed by a honey bee visit after movement from diploid (n = 34) or triploid (n = 28) male flowers. Estimated means (square mark) and 95% confidential intervals are back-transformed from the logit scale.

Across the 66 observed bee visits, 94% of bees transferred pollen (recovered on the stigma or in the storage solution), and 88% of visits resulted in at least one pollen grain germinating to the stigma. The genotype (triploid vs. diploid) of the male flower that a honey bee visited directly before visiting a triploid female flower had no significant effect on the number of pollen grains deposited on the stigma (model estimate = −0.48, SE = 0.32, *P* = 0.13, Fig. 4a). The proportion of pollen to germinate on the stigmatic surface was also not significantly different, although only marginally so (model estimate = −0.13, SE = 0.07, *P* = 0.051, Fig. 4b).

#### Pollination success

**Figure 5:**
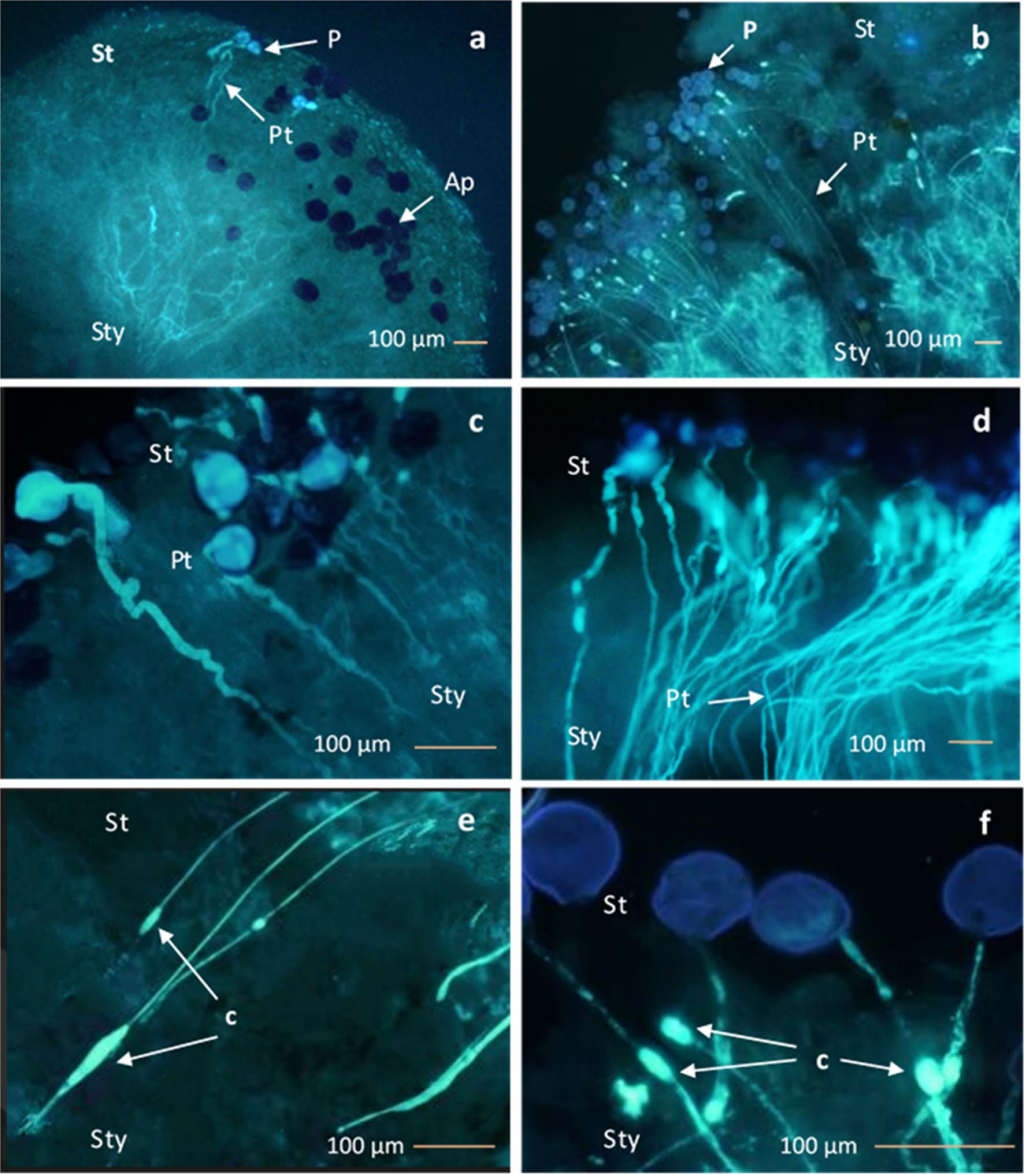
Fluorescence micrographs showing pollen tube growth and behaviour of pollen grains on pistil in two hand-pollinated crosses; triploid × triploid cross **(a,c,e)** and triploid × diploid cross **(b,d,f)**; **(a)** pollen grains (P), pollen tube (Pt) and abnormal pollen grains which were not stained (Ap) in the upper surface of stigma (St), **(c)** short twisted thick pollen tube (Pt) developed from top of the stigma (St), **(e)** irregular callose plugs developed in the pollen tubes; **(b)** stained pollen grains (P) and pollen tubes (Pt) in the pistil, **(d)** long thin pollen tubes, **(f)** regular callose plugs in the pollen tubes.

Pollen grains from both diploids and triploids germinated and produced pollen tubes on the stigmas of the triploid cultivar ‘Exclamation’ (Fig. 5a,b), however there were significant differences in the pollen germination between genotypes. In triploid × diploid crosses 31 ± 4 % of pollen germinated, compared to 6.5 ± 2 % of pollen in triploid × triploid crosses. The number of pollen tubes through the pistil also differed. The number of pollen tubes was consistently higher in all parts of the pistils when pollinated with diploid pollen (surface of stigma: model estimate = −2.98, SE = 0.07, *P* < 0.001, middle section of pistil: model estimate = −3.37, SE = 0.07, *P* < 0.001, bottom section of pistil: model estimate = −3.27, SE = 0.13, *P* < 0.001, Supplementary Fig. S5). For both crosses, the number of pollen tubes extending pass the stigmatic surface and down the pistil decreased with distance down the style. In triploid x diploid crosses 42 ± 3 % of pollen tubes on the upper surface of stigma reached to the bottom of pistil, compared to 19 ± 7 % in triploid x triploid crosses.

In the triploid × triploid crosses, only the pollen grains with regular, characteristic tricolporate shape and size (~75 μm) developed pollen tubes (Fig. 5a,c). Abnormally large pollen grains that were predominantly found in triploid × triploid crosses did not germinate (Fig. 5a). The pollen tubes resulting from triploid × triploid crosses were thick and twisted, and most of them grew only a shorter distance through the pistil. Further, deposition of callose plugs was irregular and thick (Fig. 5c,e). In contrast, the pollen tubes found in triploid × diploid crosses showed normal pollen tube development evidenced by thin walls that grew straight without twisting through the pistil and producing regular-sized callose plugs (Fig. 5d,f).

## Discussion

Knowledge of pollen characteristics and movement within and among crop plant genotypes is critical for determining whether pollen from male-sterile cultivars can interfere with the reproductive success of hybrid crops. Here we investigate whether pollen produced by triploid and diploid plants differs morphologically, how pollen from triploids interacts with receptive stigmas, and its movement in a commercial field setting.

The pollen from all watermelon flowers had several common characteristics, including having three pori, three-colpi, and exine ornamentation^20,21^; but there were also morphological differences between the diploid and triploid genotypes. These differences were consistent across cultivars, enabling us to reliably distinguish pollen in diploids and triploids. Pollen grains in triploids were polymorphic and contained a higher proportion of abnormal, larger sized pollen grains and were more abundant as tetrads compared to diploids. These differences may be the result of the formation of variants among the microspores, due to the uneven number of chromosomes (e.g. n = 33) during the first meiotic division in the pollen mother cells^22,23^. Further, the size of pollen grains has previously been correlated with the number of chromosomes in plants, another possible explanation for why pollen grains in triploids were larger^24,25^. Our findings are consistent with previous studies demonstrating that the development of pollen microspores on anthers in diploid and triploid watermelons can be abnormal^21^ and other triploid hybrids (non-crop species) having abnormal or deformed pollen grains (*Betula* species^26^ European Oak^27^ and Peonies^24^).

We show that pollen produced by triploid watermelon flowers can germinate on receptive stigmas. The likelihood of germination was however significantly lower (a sevenfold difference in our hand pollination experiment) when compared to pollen from diploids. Although low pollen germination in triploid plants has been reported^28^, the very low pollen adhesion*/*germination in triploids watermelon was unexpected because watermelon stigmas are ‘wet’, due to stigmatic secretion, and wet stigmas can typically secure a wide range of pollen types^29^. Watermelon stigmas have previously been shown to produce pollen adhering secretions not just in response to viable pollen but also in reaction to denatured pollen^30^ and heterospecific pollen^29,31^. The pollen grains in triploids may have become dislodged more easily and/or not germinated to stigmas due to their tendency to form large abnormal pollen grains and poorly developed exines. Large abnormal pollen grains (as we observed in triploids but not in diploids in this study) often cannot properly germinate with the papillae cells on the stigmatic surface^32,33^. Pollen-stigma interactions are also known to be affected by the exine layers and pollen coats^34^.

A small number (< 7%) of pollen grains from triploids (cultivar ‘Exclamation’), were able to germinate and produce pollen tubes. This suggests that some viable pollen grains can be produced by triploid cultivars, a contrast to previous reports^35,36^. However, their growth was typically restricted to the surface end of the pistil, with fewer than 20% of the germinating pollen grains reaching the bottom of the pistil. Their tubes were also thick and twisted in appearance with irregular callose plugs. The latter is characteristic of self-incompatibility; a mechanism in flowering plants that prevents inbreeding and promotes outcrossing^37–39^. Thus, whilst we observed pollen grains from triploids that were normal in appearance, the majority of pollen from the assessed triploid cultivars was classified as abnormal and > 90% did not germinate on viable stigmas. This is an indication that pollen from triploids was mostly functionally inviable -the growth of any viable pollen grains was typically blocked before fertilisation could take place.

We also assessed pollen movement by honey bees among inter-planted cultivars. This was achieved by comparing single visit pollen transfer onto test stigmas after a visit to either a male diploid or triploid flower. Eighty eight percent of bee visits resulted in at least one pollen grain germinating on the stigma, but in contrast to our hand-pollination study, we did not find a significant difference in pollen germination with genotype. This is likely to be because honey bees visit both genotypes while foraging^17,40^, which will have resulted in a number of viable pollen grains from diploids being deposited on our stigmas even if a bee had moved directly from a triploid male flower. We also found that the proportion of bee transferred pollen to germinate to the stigma was higher for both genotypes compared to our hand-pollinations. The simplest explanation is that the honey bees were more effective pollinators^41^, however it is possible that a greater number of pollen grains from diploids germinated as the result of recipient interaction with pollen from triploids in the stigma, a mechanism has previously been considered for heterospecific pollen (e.g., a heard effect^42^). The potential for interaction (neutral/positive/negative) among the pollen genotypes was not assessed in the current study, but the morphological descriptions we provide will permit other researchers to conduct this comparison.

In summary, we show that there are consistent morphological and physiological differences in pollen produced by diploid and triploid watermelon cultivars. The distinguishing features of pollen in triploids included a higher number of deformities, tetrads, and abnormal growth of callose plugs. When applied alone, some pollen in triploids was able to geminate on viable stigmas, but germination and the rate of pollen tube growth was consistently very low. So whilst it is likely that honey bees will collect and deposit some viable pollen from triploids onto stigmas, its low germination suggests that there is minimal likelihood of it preventing fertilisation by diploid pollen, either by mechanical clogging and/or inhibition of pollen tube growth.

## Methods

### Model hybrid crop species

Watermelon (*Citrullus lanatus* (Thunb.) Matsum. & Nakai); family: Cucurbitaceae) is an economically important food crop (global economic value of US $ 27.9 × 10^3^ M^43^) that depends entirely on insect pollination for fruit set^44^. Watermelon (originally diploid: 2n = 22) has a monoecious flowering habit producing separate male and female flowers on the same plant^45^. Seedless watermelon (triploid hybrids, 3n = 33) are produced by crossing a diploid and a tetraploid parent, and are now the dominant cultivar in major watermelon growing areas such as North America, where over 80% of watermelons are seedless^46,47^. Whilst diploid cultivars have viable, self-compatible pollen^29^ and do not require cross-pollination^48^, triploid cultivars, with their three sets of chromosomes, do not produce viable pollen (male gametes) and need to be inter-planted with pollen-donating diploids^49^.

Three common diploid cultivars (‘SP-6’, ‘Summer Flavor 800’ and ‘Tiger’) and three triploid cultivars (‘Javelin’, ‘Exclamation’ and ‘Royal Armada’) were used in the current study. Two commercial melon farms at Chinchilla, QLD Australia (location: 26°0′52.44′′S, 150°7′5.64′′E, average temperature: 25.3 °C) and another two farms at Riverina, NSW, Australia (location: 34°11′13′′S, 146°2′42′′E, average temperature: 22.6 °C), were selected for the study. The farms followed in-row planting system with 3:1 ratio of seedless and polliniser cultivars (including one triploid cultivar and one or more diploid cultivars per block: Chinchilla block 1: ‘Exclamation’ and ‘SP-6’; Chinchilla block 2: ‘Javelin’ and ‘Tiger’; Riverina block 1: ‘Royal Armada’ and ‘Tiger’; Riverina block 2: ‘Royal Armada’ and ‘Summer Flavor 800’) and were maintained according to recommended cultural and management practices. Crop plant material was sampled and processed with permission from farm owners and specimens are housed at the University of New England and Plant and Food Research, Australia. All relevant permissions were obtained to use commercial farms sampled in this study. Further, all aspects of this study were conducted in compliance with institutional, national and international regulations.

### Pollen morphology

To determine whether there are differences in the morphology of the pollen produced by triploid (seedless) and diploid (seeded) genotypes, we compared pollen from 5/6 of the cultivars listed above (not ‘Javelin’). To obtain pollen, six randomly selected male flowers from each cultivar were bagged while still in bud. The flowers were then collected the following day after anthesis, and the anthers were excised and stored in EtoH. The pollen from each flower were prepared separately using acetolysis^50^, then stained with 1% fuschin, and mounted on microscopic slides using glycerine^51^. Five pollen slides were produced from each flower to produce 150 slides across all cultivars. Pollen grains were then categorized and counted using a light microscope (Nikon 90i Brightfield Dic S002) and imaging software (NIS-Elements Viewer Ver4.50.00) on the same or following day, to minimise any effect the mounting medium might have on the pollen^52^. A total of 31,890 pollen grains were observed across the five cultivars. To categorize the pollen for each flower of each cultivar, we measured the surface area, perimeter and polar axis (P) (in polar view), equatorial diameter (E), colpi length and colpi width, pori length, and pori width (in equator view), the polar axis/equatorial diameter (P/E) and colpi length /polar axis in randomly selected pollen grains (Supplementary Fig. S1). We also checked non-acetolyzsed pollen grains from both cultivars under a light microscope, to confirm the preparation method had not significantly affected pollen morphology (Supplementary Fig. S6). We followed the palynological terminology given in reference materials^53–55^.

### Pollen-pistil interaction within a field: triploid × diploid and triploid × triploid crosses

#### Hand-pollination

To determine whether there are differences in the pollen-pistil interaction with genotype cross, triploid female flowers were hand pollinated with pollen from diploid or triploid plants grown within the same field and subsequent pollen tube growth compared. Randomly selected 16 virgin female flowers were bagged as buds across eight plants (two buds per plant) of cultivar ‘Exclamation’. To obtain pollen, 16 male flower buds on inter-planted triploids (cultivar ‘Exclamation’) and diploids (cultivar SP-6) were bagged separately on 16 different plants before anthesis. These flowers were harvested after confirming anthers had dehisced and released pollen grains—pollen visible when touched. To pollinate the female flowers, a separate dehisced anther was gently brushed against each of the three lobes of the stigma in each flower (Supplementary Fig. S7). To ensure maximum receptivity, the eight triploid x diploid and eight triploid x triploid hand pollination crosses were performed between 6:00 a.m. to 10:00 a.m.^48^. The pollinated female flowers were then exercised, their stems wrapped in moist tissue paper and the whole flower carefully placed in individual zip-lock bags for 24 h to allow the pollen to germinate^56^. The flowers’ pistils (only stigma and style) were subsequently exercise and stored in 1.5 ml Eppendorf tubes containing 70% ethanol solution until processed.

To assess pollen germination to the stigma, each pistil was softened in 8 M NaOH for 24 h (soaking time yielded optimal mounting of stigmas). The pistils were subsequently rinsed in deionised water and stained with 0.1% aniline blue in 0.1% K_2_HPO_4_ and mounted on slides^57^. Pollen grains on the stigma surface were counted under UV light. We assumed that the pollen in the solutions detached from the stigmas because it had not germinated. Ten subsamples from both NaOH and EtoH solutions for each stigma were checked for pollen using a haemocytometer (Bright-Line™, Cambridge instruments)^58^. The number pollen germinated on the stigma and suspended in the stored solutions were totalled to give pollen transfer from anther to stigma in each cross.

#### Pollen deposition by honey bees

To assess field-realistic pollen-pistil interactions, we determined the number of pollen grains that germinate on stigmas after a honey bee visited either a triploid or a diploid male flower. Previously bagged female flowers (from triploid cultivars ‘Exclamation’ and ‘Javelin’) were removed and presented to honey bees on marked male flowers^59^ of a known genotype (diploid cultivars ‘SP-6’ and ‘Tiger’). Once a honey bee moved onto the treatment flower, it was allowed to visit the flower uninterrupted. All flower visits took place between 8:00 a.m. and 11:30 a.m. Visited stigmas (n = 66) were processed and pollen grains counted on the stigmatic surface^60^, and storage solutions as outlined above.

### Measuring pollination success

**Figure 6:**
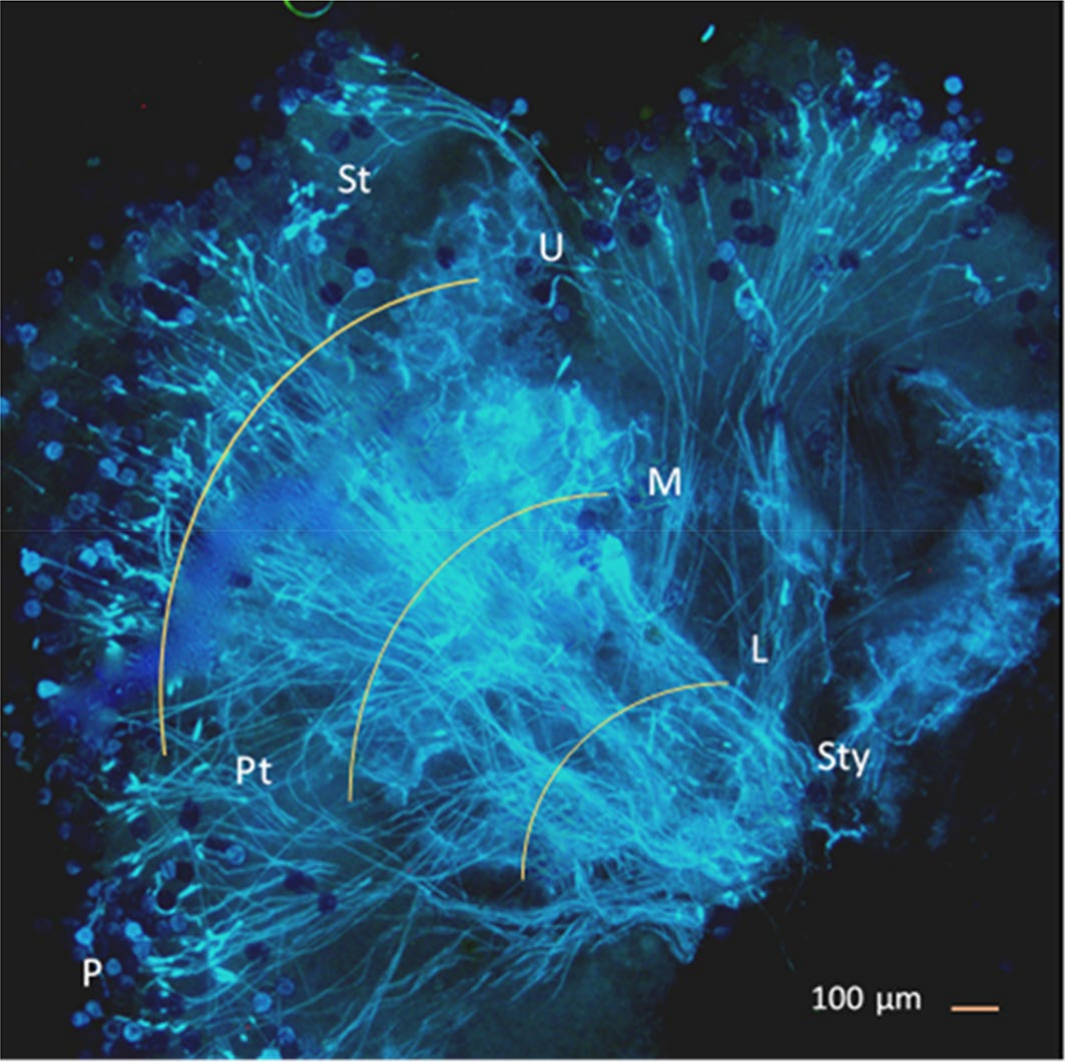
A fluorescence micrograph of a pistil showing pollen grains (P) on the stigma (St), and pollen tubes (Pt) in the upper (U), middle (M) and lower (L) 1/3 of the of style (Sty).

To estimate pollination success of pollen in diploid and triploid cultivars, we recorded the number of pollen tubes present in our hand-pollinated stigmas, using a fluorescence microscope under UV light^61^. For each stigma, we counted the number of pollen grains on the surface of the stigma, and the number of pollen tubes present in a transect in the upper, middle, and lower 1/3 section of the style at 40× magnification (Fig. 6).

### Statistical analysis

All statistical analysis were conducted using R statistical software^62^. First, we compared the percentages of symmetrical pollen grains as monads and tetrads, and recorded pollen abnormalities, between genotypes and among cultivars. We used a linear mixed effect model (package nlme^63^) for each trait (including: surface area, perimeter, P, E, P/E, colpi length, colpi width, pori length, pori width and colpi length/polar axis) separately, to determine whether they differed among cultivars and between genotypes. Cultivar and genotype were included as fixed factors and flower ID as a random factor. Mean separation tests were conducted to compare traits among cultivars and genotypes (package emmeans^64^).

The number of pollen grains transferred from anthers to stigmas, number of pollen in solutions, and number of pollen tubes in different positions of pistil (top, middle and lower) were compared for triploid × triploid and triploid × diploid crosses, using generalized linear mixed effect models (GLMMs) with a Poisson distribution (package glmmTMB^65^). Another GLMM model with binomial error distribution^65^ was used to compare the proportion of pollen adhering over total pollen transfer to stigma between hand pollinated cross. Type of hand pollinated cross was included as the fixed effect while stigma ID was included as a random effect in both models.

A GLMM with a negative binomial error distribution^65^ was used to compare the pollen germinating on stigmas after a honey bee moves from diploid or triploid male flower. Number of pollen germinate to stigma was the response variable. Another GLMM model with binomial error distribution^65^ was used to compare the proportion of pollen adhering over total pollen transfer to triploid stigma after a honey bee moves from diploid or triploid male flower. Male flower type were fixed effect while site was a random effect in both models. Post-hoc analysis was carried out using package emmeans^64^. We performed model diagnostics and validated the fit of all models with the DHARMa package^66^.

## Supplementary Information

Supplementary Information.

## Data Availability

The datasets generated and analysed during the study is available from the Dryad Digital Repository. https://doi.org/10.5061/dryad.v15dv41xg
