# Supplementary material for: Morphological characteristics of pollen from triploid watermelon and its fate on stigmas in a hybrid crop production system
Source: Sci Rep. 2022 Feb 25;12:3222. doi: 10.1038/s41598-022-06297-2 (PMC8881623; doi:10.1038/s41598-022-06297-2)
Supplement: Supplementary file 1 — Supplementary Information. [file 41598_2022_6297_MOESM1_ESM.pdf]

Morphological characteristics of pollen from triploid watermelon and its fate on stigmas in a hybrid crop production system

Erandi C. W. Subasinghe Arachchige, Lisa J. Evans, Ulrika Samnegård and Romina Rader

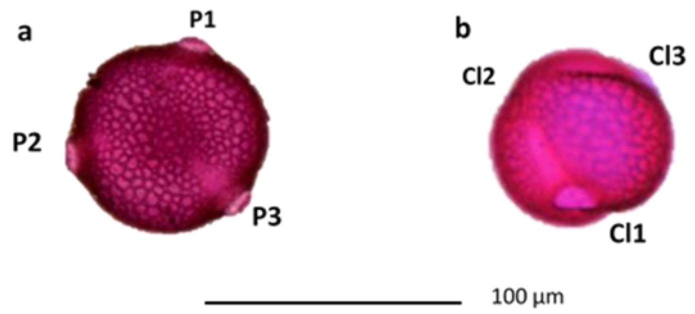

Supplementary Fig. S1: Images of tricolporate pollen grains of diploid cultivar 'Summer Flavor 800' with different pollen apertures a) polar view of a pollen grain showing three pori (P1, P2 and P3) b) a pollen grain showing three colpi (Cl1, Cl2 and Cl3).

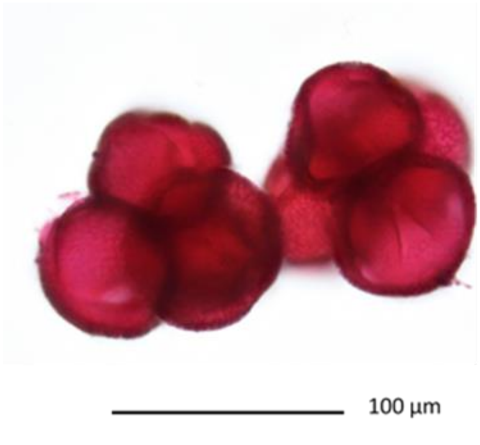

Supplementary Fig. S2: Image of a tetrad found in triploids.

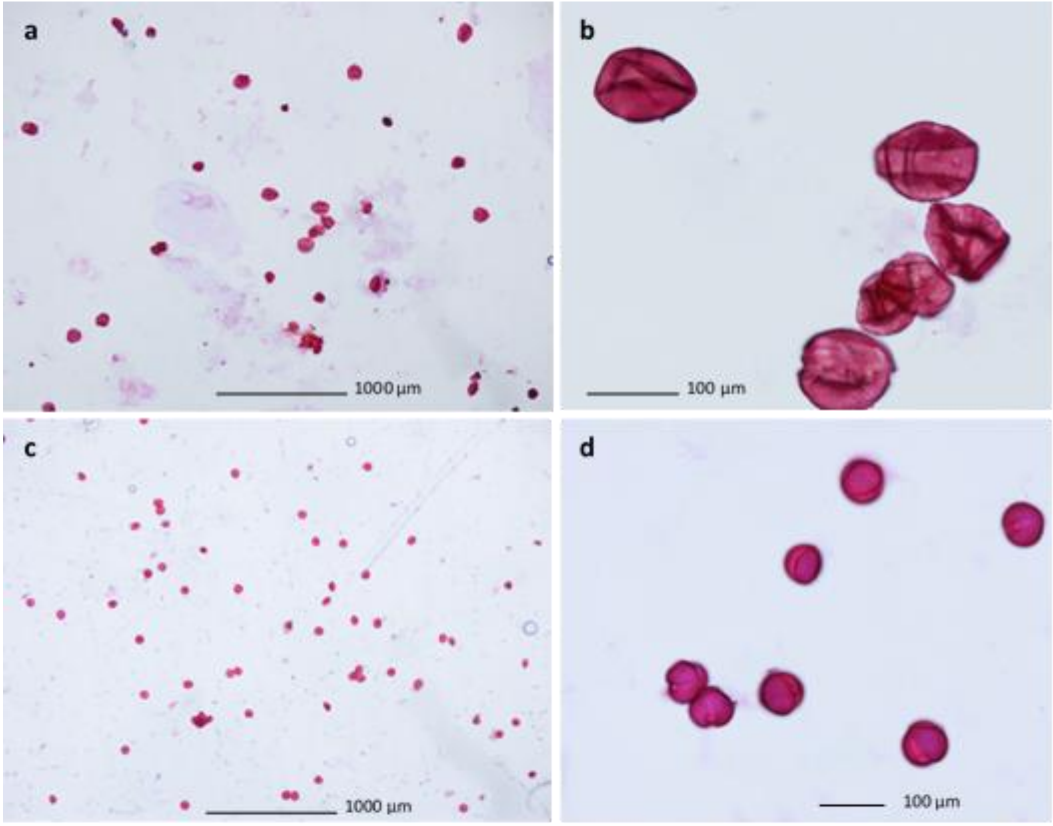

Supplementary Fig. S3: Micrographs showing pollen grains in triploid cultivar 'Exclamation' (a and b) and diploid cultivar 'SP-6' in watermelon (c and d).

Supplementary Table. S1: Summary of Analysis of variance (ANOVA) for pollen morphological traits in cultivars and genotypes resulted from linear mixed effect models

**Response**	**numDF**	**denDF**	**F value**	***P*-value**
**Cultivar**				
Surface area				
(Intercept)	1	330	7473.83	<.0001
Cultivars	4	25	32.26	<.0001
Perimeter				
(Intercept)	1	330	26974.05	<.0001
Cultivars	4	25	26.18	<.0001
Equator diameter				
(Intercept)	1	330	31240.87	<.0001
Cultivars	4	25	34.65	<.0001
Polar axis				
(Intercept)	1	330	27989.29	<.0001
Cultivars	4	25	28.78	<.0001
P/E				
(Intercept)	1	330	15970.65	<.0001
Cultivars	4	25	0.51	0.73
Pori length				
(Intercept)	1	330	7694.29	<.0001
Cultivars	4	25	16.61	<.0001
Pori width				
(Intercept)	1	330	6264.98	<.0001
Cultivars	4	25	47.93	<.0001
Colpi length				
(Intercept)	1	330	15233.54	<.0001
Cultivars	4	25	10.79	<.0001
Colpi width				
(Intercept)	1	330	6108.56	<.0001
Cultivars	4	25	0.77	0.55
Colpi length / Polar axis				
(Intercept)	1	330	14962.32	<.0001
Cultivars	4	25	0.81	0.53
**Genotype**				
Surface area				
(Intercept)	1	330	1636.96	<.0001
Genotypes	1	28	5.94	0.02
Perimeter				
(Intercept)	1	330	7048.66	<.0001

Genotypes	1	28	6.91	0.01
Equator diameter				
(Intercept)	1	330	6315.00	<.0001
Genotypes	1	28	5.64	0.02
Polar axis				
(Intercept)	1	330	6200.03	<.0001
Genotypes	1	28	3.07	0.09
P/E				
(Intercept)	1	330	16920.66	<.0001
Genotypes	1	28	0.48	0.49
Pori length				
(Intercept)	1	330	2532.76	<.0001
Genotypes	1	28	3.83	0.06
Pori width				
(Intercept)	1	330	1041.85	<.0001
Genotypes	1	28	6.53	0.02
Colpi length				
(Intercept)	1	330	6117.38	<.0001
Genotypes	1	28	2.68	0.11
Colpi width				
(Intercept)	1	330	6073.45	<.0001
Genotypes	1	28	0.45	0.50
Colpi length / Polar axis				
(Intercept)	1	330	14437.52	<.0001
Genotypes	1	28	0.07	0.79

Supplementary Table S2: Statistical output obtained from linear mixed effect models for pollen morphological traits

	Value	SE	DF	t-value	*P*-value
**Cultivar**					
**Surface area** (µm^2)					
(Intercept)	4507.34	113.74	330	39.63	0.00
variety royal armada	-1010.03	160.85	25	-6.27	0.00
varietySP-6	-1289.83	142.89	25	-9.02	0.00
varietysummerflavor	-1217.83	142.89	25	-8.52	0.00
varietytiger	-397.63	142.89	25	-2.78	0.01
**Perimeter** (µm)					
(Intercept)	248.65	3.75	330	66.30	0.00
variety royal armada	-29.66	5.30	25	-5.59	0.00
varietySP-6	-37.80	4.64	25	-8.14	0.00
varietysummerflavor	-37.30	4.64	25	-8.03	0.00
varietytiger	-13.43	4.64	25	-2.89	0.01
**Equator diameter** (µm)					
(Intercept)	75.39	1.02	330	73.23	0.00
variety royal armada	-9.13	1.45	25	-6.26	0.00
varietySP-6	-11.72	1.29	25	-9.06	0.00
varietysummerflavor	-11.36	1.29	25	-8.79	0.00
varietytiger	-3.26	1.29	25	-2.52	0.02
**Polar axis** (µm)					
(Intercept)	71.75	1.04	330	68.49	0.00
variety royal armada	-6.51	1.48	25	-4.39	0.00
varietySP-6	-9.42	1.33	25	-7.10	0.00
varietysummerflavor	-9.29	1.33	25	-7.07	0.00
varietytiger	-0.26	1.33	25	-0.19	0.84
**Polar axis / Equator diameter**					
(Intercept)	0.96	0.02	330	45.64	0.00
variety royal armada	0.03	0.03	25	0.93	0.36
varietySP-6	0.02	0.03	25	0.81	0.42
varietysummerflavor	0.02	0.03	25	0.72	0.48
varietytiger	0.04	0.03	25	1.38	0.17
**Pori length** (µm)					
(Intercept)	13.39	0.40	330	33.25	0.00
variety royal armada	-2.02	0.57	25	-3.55	0.00
varietySP-6	-2.78	0.48	25	-5.81	0.00
varietysummerflavor	2.60	0.48	25	-5.43	0.00
varietytiger	-0.58	0.48	25	-1.21	0.23

**Pori width** (µm)					
(Intercept)	15.63	0.46	330	33.79	0.00
variety royal armada	-2.98	0.65	25	-4.55	0.00
varietySP-6	-5.43	0.56	25	-9.72	0.00
varietysummerflavour	-4.75	0.56	25	-8.47	0.00
varietytiger	-0.60	0.56	25	-0.99	0.29
**Colpi length**(µm)					
(Intercept)	58.63	1.30	330	45.00	0.00
variety royal armada	-5.51	1.84	25	-2.99	0.01
varietySP-6	-7.66	1.56	25	-4.91	0.00
varietysummerflavor	-6.42	1.56	25	-4.11	0.00
varietytiger	-1.49	1.56	25	-0.95	0.37
**Colpi width** (µm)					
(Intercept)	10.93	0.42	330	26.22	0.00
variety royal armada	-0.08	0.59	25	-0.13	0.89
varietySP-6	-0.60	0.49	25	-1.23	0.23
varietysummerflavor	-0.20	0.49	25	-0.32	0.75
varietytiger	-0.02	0.49	25	-0.04	0.96
**Colpi length/ Polar axis**					
(Intercept)	0.82	0.02	330	40.63	0.00
variety royal armada	0.00	0.03	25	-0.02	0.98
varietySP-6	0.01	0.02	25	0.28	0.78
varietysummerflavor	0.02	0.02	25	0.78	0.43
varietytiger	-0.01	0.02	25	-0.57	0.56
**Genotype**					
**Surface area** (μm^2)					
(Intercept)	3539.21	115.49	330	30.64	0.00
Genotype triploid	463.11	189.99	28	2.43	0.02
**Perimeter**(µm)					
(Intercept)	219.16	3.34	330	65.72	0.00
Genotype triploid	14.66	5.57	28	2.62	0.01
**Equator diameter**(µm)					
(Intercept)	66.61	1.08	330	61.56	0.00
Genotype triploid	4.21	1.77	28	2.37	0.02
**Polar axis**(µm)					
(Intercept)	65.43	1.06	330	61.35	0.00
Genotype triploid	3.06	1.75	28	1.75	0.09
**Polar axis / Equator diameter**					
(Intercept)	0.99	0.01	330	111.69	0.00
Genotype triploid	-0.01	0.02	28	-0.68	0.50

**Pori length** (µm)					
(Intercept)	11.41	0.28	330	40.29	0.00
Genotype triploid	0.96	0.49	28	1.96	0.06
**Pori width** (µm)					
(Intercept)	12.04	0.49	330	24.16	0.00
Genotype triploid	2.11	0.82	28	2.55	0.02
**Colpi length** (µm)					
(Intercept)	53.43	0.83	330	63.81	0.00
Genotype triploid	2.44	1.49	28	1.64	0.11
**Colpi width** (µm)					
(Intercept)	10.67	0.15	330	68.64	0.00
Genotype triploid	0.22	0.33	28	0.66	0.50
**Colpi length/ Polar length**					
(Intercept)	0.82	0.01	330	105.76	0.00
Genotype triploid	-0.004	0.02	28	-0.26	0.79

Linear mixed effect models were performed with pollen morphological traits as the dependent variables, cultivars and genotypes as fixed effects and flower ID used to obtain pollen was as the random factor. Summary tables for analyses including model-predicted values, their standard errors (SE), degrees of freedom (DF), t values, and *P* values.

Supplementary Table. S3: Pollen morphological traits (mean ±SE) in different watermelon cultivars

Cultivar	Surface area (μm^2)	Perimeter (µm)	Equator diameter (µm)	Polar axis (µm)	Polar axis/ equator diameter	Pori length (µm)	Pori width (µm)	Colpi length (µm)	Colpi width (µm)	Colpi length/Polar axis
Exclamation	4489±167^{b}	248±4.63^{b}	75.2±1.34^{b}	71.9±0.86^{b}	0.96±0.02^{a}	13.3±0.41^{b}	15.6±0.41^{c}	58.6±1.36^{c}	11±0.34^{a}	0.82±0.02^{a}
Royal Armada	3497±72.9^{a}	219±2.43^{a}	66.3±0.76^{a}	65.2±0.71^{a}	0.99±0.05^{a}	11.4±0.28^{a}	12.7±0.39^{b}	53.1±0.78^{ab}	10.9±0.42^{a}	0.82±0.01^{a}
SP-6	3218±31.3^{a}	211±1.21^{a}	63.7±0.31^{a}	62.3±0.41^{a}	0.98±0.01^{a}	10.6±0.18^{a}	10.2±0.20^{a}	51±0.43^{a}	10.3±0.21^{a}	0.82±0.01^{a}
Summer Flavor 800	3288±40.3^{a}	211±1.85^{a}	64±0.42^{a}	62.5±0.40^{a}	0.98±0.01^{a}	10.8±0.18^{a}	10.9±0.20^{a}	52.2±0.65^{a}	10.8±0.20^{a}	0.84±0.01^{a}
Tiger	4108±61.1^{b}	235±1.91^{b}	72.1±0.54^{b}	71.5±0.56^{b}	0.99±0.01^{a}	12.8±0.25^{b}	15.2±0.28^{c}	57.1±0.83^{bc}	10.9±0.23^{a}	0.80±0.01^{a}
P-value	<0.001	<0.001	<0.001	<0.001	0.73	<0.001	<0.001	<0.001	0.55	0.53

Note: The different lower-case letters indicate the significant differences among cultivars based on pairwise comparison of estimated marginal means of pollen morphological traits (Tukey-adjusted pairwise comparison at α = 0.05).

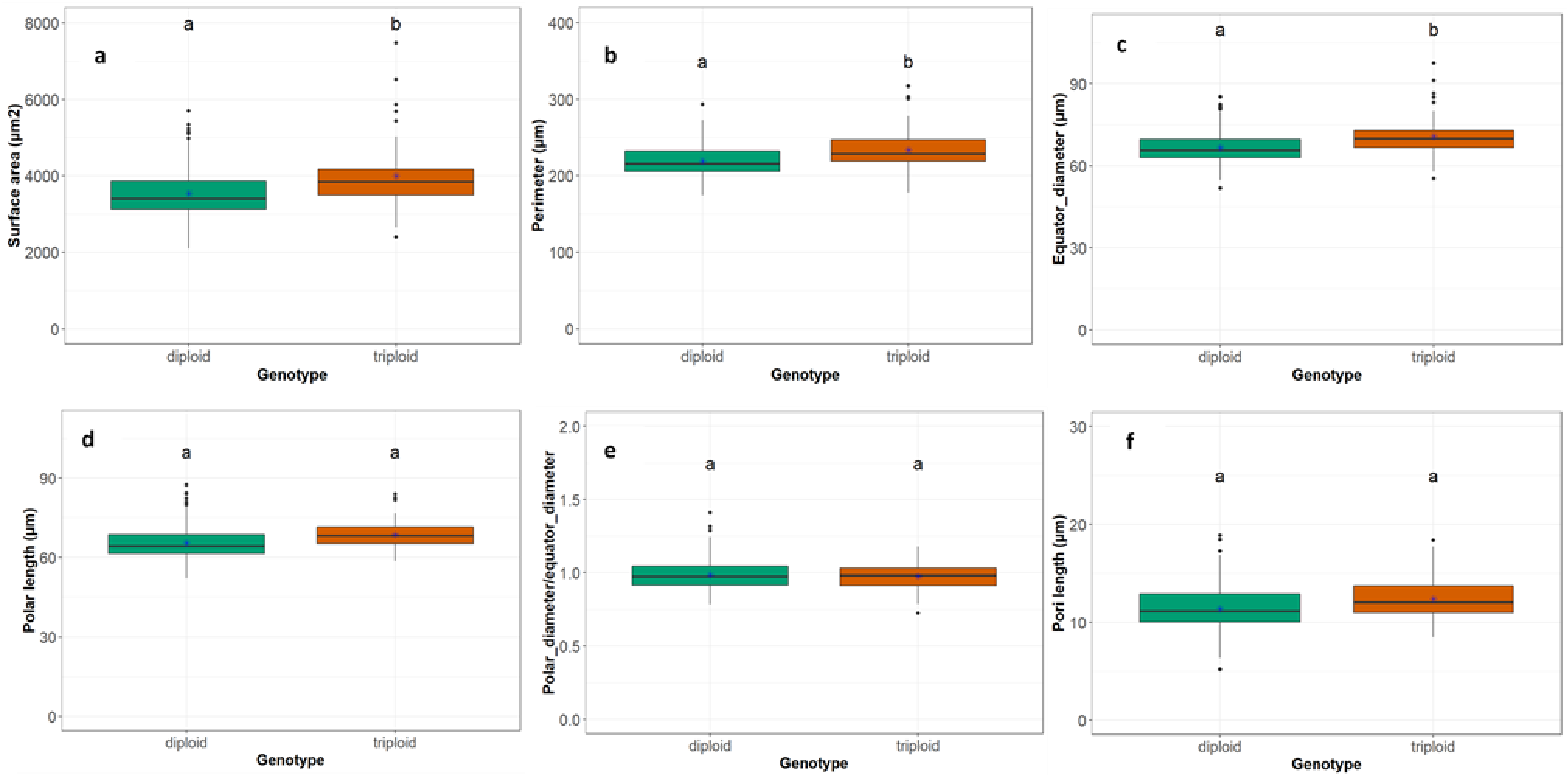

a
Surface area (µm2)
8000
6000
4000
2000
0
a
b
diploid
triploid
Genotype
b
Perimeter (µm)
400
300
200
100
0
a
b
diploid
triploid
Genotype
c
Equator_diameter (µm)
90
60
30
0
a
b
diploid
triploid
Genotype
d
Polar length (µm)
90
60
30
0
a
a
diploid
triploid
Genotype
e
Polar_diameter/equator_diameter
2.0
1.5
1.0
0.5
0.0
a
a
diploid
triploid
Genotype
f
Pori length (µm)
30
20
10
0
a
a
diploid
triploid
Genotype

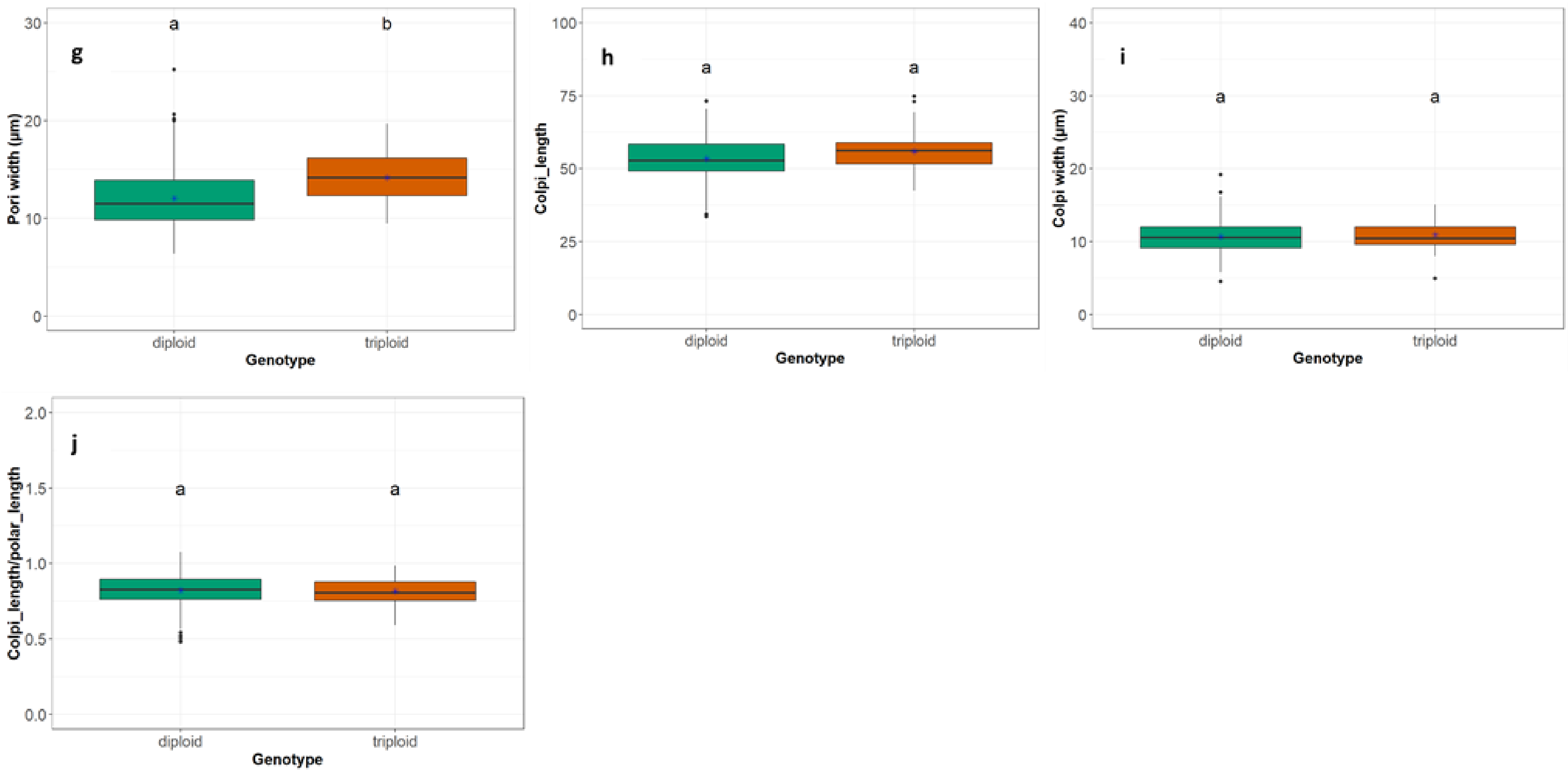

Supplementary Fig. S4: Boxplots showing of different pollen traits in two watermelon genotypes (a-j); Box indicates quartiles with median and points outside of the box are outliers, mean values are marked as asterisk (*). The different lower-case letters indicate the significant differences between genotypes for each pollen morphological traits (EMMeans pairwise comparisons at $\alpha = 0.05$).

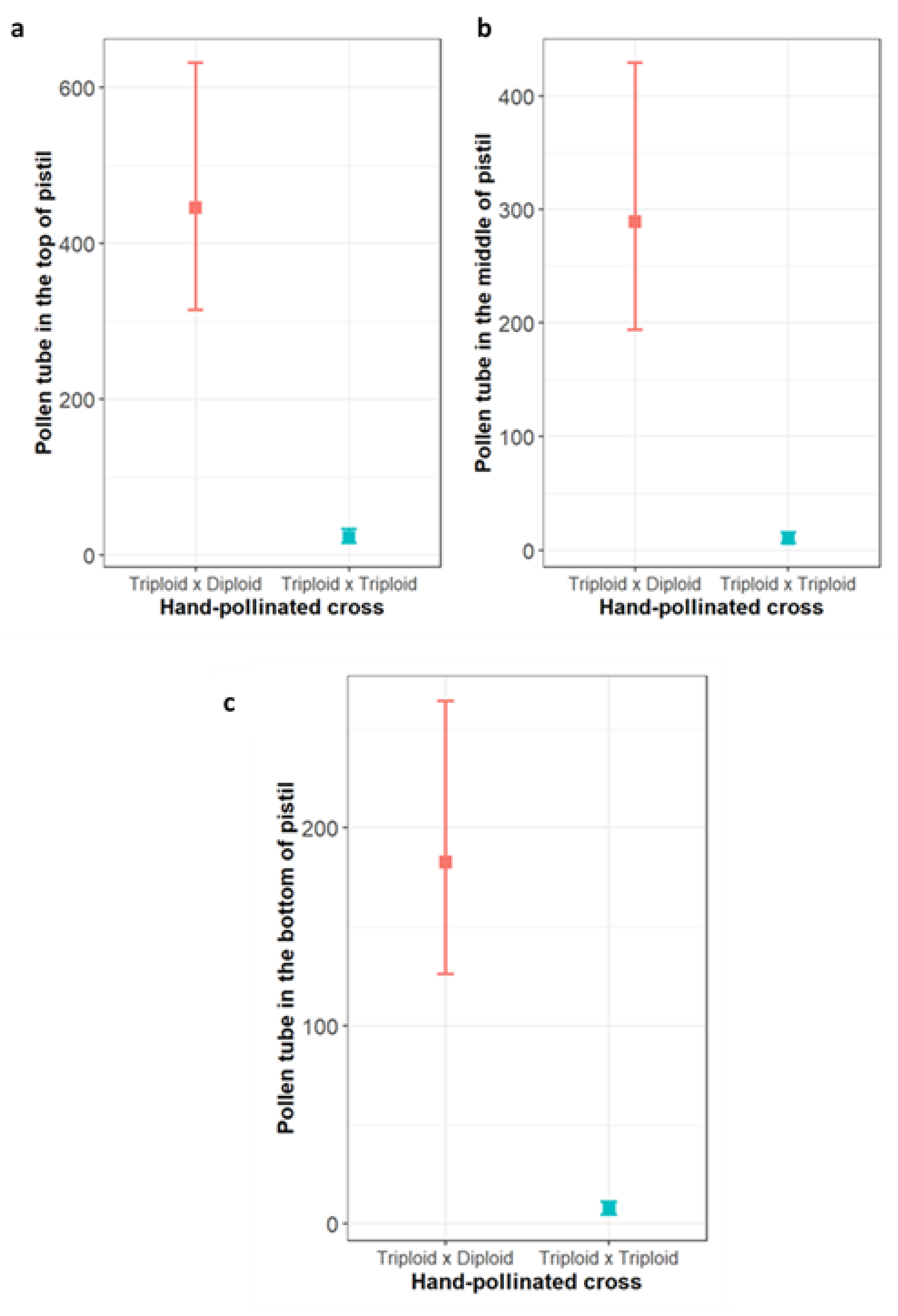

Supplementary Fig. S5: Number of pollen tubes observed in the (a) top, (b) middle and (c) bottom of the style in two hand-pollinated crosses; triploid x triploid cross (n=8) and triploid x diploid cross (n=8). Estimated means (square mark) and 95% confidential Intervals are back-transformed from the logit scale.

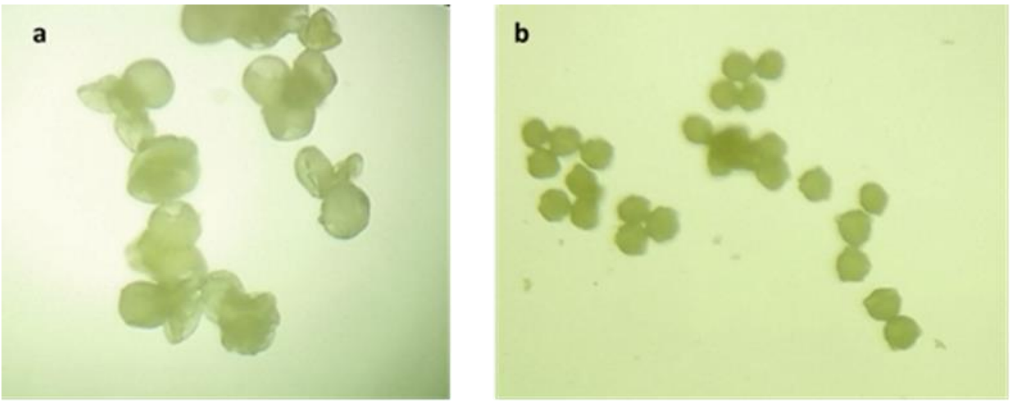

Supplementary Fig. S6: Micrographs showing non-acetolysed pollen grains in a) triploids and b) diploids.

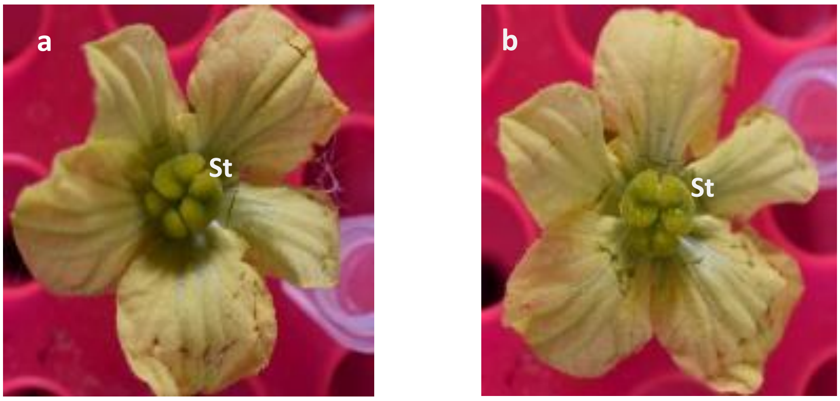

Supplementary Fig. S7: Images of female flower showing A) receptive wet stigma (St) before pollination; B) dried stigma (St) with pollen grains after pollination.
